# Metabolomics of small extracellular vesicles derived from isocitrate dehydrogenase 1-mutant HCT116 cells collected by semi-automated size exclusion chromatography

**DOI:** 10.3389/fmolb.2022.1049402

**Published:** 2023-01-11

**Authors:** Ryosuke Hayasaka, Sho Tabata, Masako Hasebe, Satsuki Ikeda, Tomoya Hikita, Chitose Oneyama, Jun Yoshitake, Daisuke Onoshima, Kumiko Takahashi, Takahiro Shibata, Koji Uchida, Yoshinobu Baba, Tomoyoshi Soga, Masaru Tomita, Akiyoshi Hirayama

**Affiliations:** ^1^ Institute for Advanced Biosciences, Keio University, Tsuruoka, Japan; ^2^ Systems Biology Program, Graduate School of Media and Governance, Keio University, Fujisawa, Japan; ^3^ Institute for Protein Research, Osaka University, Suita, Japan; ^4^ Division of Cancer Cell Regulation, Aichi Cancer Center Research Institute, Nagoya, Japan; ^5^ Department of Oncology, Graduate School of Pharmaceutical Sciences, Nagoya City University, Nagoya, Japan; ^6^ Department of Target and Drug Discovery, Graduate School of Medicine, Nagoya University, Nagoya, Japan; ^7^ Institute of Nano-Life-Systems, Institutes of Innovation for Future Society, Nagoya University, Nagoya, Japan; ^8^ Materials Integration Laboratories, AGC Inc., Yokohama, Japan; ^9^ Institute for Glyco-core Research (iGCORE), Nagoya University, Nagoya, Japan; ^10^ Graduate School of Agricultural and Life Sciences, The University of Tokyo, Tokyo, Japan; ^11^ Japan Agency for Medical Research and Development, CREST, Tokyo, Japan; ^12^ Department of Biomolecular Engineering, Graduate School of Engineering, Nagoya University, Nagoya, Japan; ^13^ Institute of Quantum Life Science, National Institutes for Quantum and Radiological Science and Technology, Chiba, Japan; ^14^ College of Pharmacy, Kaohsiung Medical University, Kaohsiung, Taiwan

**Keywords:** small extracellular vesicles, size exclusion chromatography, ultracentrifugation, metabolome analysis, lipidome analysis, 2-hydroxyglutaric acid, oncometabolites

## Abstract

Cancer-derived small extracellular vesicles (sEVs) are multifunctional particles with a lipid bilayer structure that are involved in cancer progression, such as malignant proliferation, distant metastasis, and cancer immunity evasion. The separation protocol used to isolate sEVs is an important process and thus, several have been developed, including ultracentrifugation (UC), size exclusion chromatography (SEC), and affinity purification using antibodies against sEV surface antigens. However, the effects of different separation methods on sEV components have not been adequately examined. Here, we developed a semi-automated system for collecting sEVs by combining SEC and preparative high-performance liquid chromatography and applied it to metabolome analysis. The developed SEC system could recover sEVs more efficiently and non-destructively than UC, suggesting that it is an appropriate recovery method for metabolic analysis and reflects biological conditions. Furthermore, using the developed SEC system, we performed metabolome analysis of sEVs from isocitrate dehydrogenase 1 (IDH)-mutated human colon HCT116 cells, which produce the oncogenic metabolite, 2-hydroxyglutaric acid (2-HG). IDH1-mutated HCT116 cells released significantly more sEVs than wild-type (WT) cells. The metabolomic profiles of IDH1 mutant and WT cells showed distinct differences between the cells and their sEVs. Notably, in IDH mutant cells, large amounts of 2-HG were detected not only in cells, but also in sEVs. These results indicate that the SEC system we developed has wide potential applications in sEVs research.

## 1 Introduction

Extracellular vesicles (EVs) are lipid bilayer structures released by various cells (Théry et al., 2018). EVs are observed in body fluids, such as urine, blood, saliva, and culture supernatants from cultured cells (Raposo and Stoorvogel, 2013). It is not possible to distinguish between exosomes (50–150 nm), microvesicles (100–1,000 nm), and apoptotic vesicles (100–1,000 nm), which all have a lipid bilayer structure. Therefore, the Internal Society of Extracellular Vesicles (ISEV) recommends that these vesicles should be called EVs (Théry et al., 2018). Small EVs (sEVs) contain functional molecules, such as DNA, mRNA, miRNA, proteins, lipids, and metabolites (Kreimer et al., 2015; Momen-Heravi et al., 2018; Williams et al., 2019; Skotland et al., 2020). Because the components in sEVs are altered by cancer and other diseases, they have been extensively studied (Steinbichler et al., 2017; Shah et al., 2018). In many cell types, tetraspanins, such as CD9, CD63, and CD81, as well as Alix and Syntenin-1, which are involved in late endosomes, are more enriched in sEVs than in cells (Van Niel et al., 2018) and therefore, have been used as sEV markers (Jeppesen et al., 2019). In recent years, several studies have been conducted on the hydrophilic metabolites in sEVs (Zhao et al., 2016; Ludwig et al., 2020; Tadokoro et al., 2020; Hayasaka et al., 2021). However, it is unclear which metabolites are contained in sEVs and whether an increase in a given metabolite in the cell is reflected in its hydrophilic metabolite content in the sEVs.

Ultracentrifugation (UC) is the gold standard for sEV recovery; however, it is often compromised by the co-precipitation and aggregation of proteins with sEVs (Nordin et al., 2015) and rupture of sEVs (Guan et al., 2020). In addition, the sample volume must be appropriate for the ultracentrifuge rotor used (e.g., 3.5 mL for an SW41Ti rotor). When the volume of the liquid is small, dilution with PBS is necessary, leading to a lower recovery rate. To overcome the limitations of current methods, the development of an easy method of recovery that does not require expensive equipment and inability to recover sEVs from multiple samples would greatly improve the study of sEVs.

In addition to UC, other recovery methods for sEVs include density gradient centrifugation, the down pellet approach, and affinity purification utilizing antibodies (Taylor and Shah, 2015). Some current obstacles include variation in the purification and recovery rates among collection methods (Patel et al., 2019; Brennan et al., 2020) and undisclosed constituents of the recovery buffers used in commercial kits. The latter is particularly challenging to sEV isolation for metabolomic and proteomic analyses because ion suppression and column failure occur when samples are prepared in buffers with a high salt content or surfactants. In the worst case, the mass spectrometer is damaged, making stable measurements impossible. Therefore, it was necessary to use a solution with a known buffer composition.

Recently, the recovery of sEVs using size exclusion chromatography (SEC) has attracted much attention (Sidhom et al., 2020). SEC is a method of isolation that is based on the difference between the time required for the migration of molecules based on their size, which can efficiently recover fractions containing sEVs that are highly pure (Baranyai et al., 2015). sEVs collection kits using SEC, such as qEV (Izon Science), EV Second (GL Sciences Inc.), and PURE-EV (HansaBioMed), have been used in previous studies (Kitamura et al., 2018; Alameldin et al., 2021; Du et al., 2021), but are limited in their versatility, automation, and multi-specimen processing. Although some studies have used SEC to collect sEVs for metabolomic analysis, the effect of different EV collection methods on metabolites is unknown.

Cancer cells metabolize differently from normal cells, including the Warburg effect, in which glycolysis is used to make ATP in an oxygen-rich environment (Warburg, 1956). Some metabolites, called oncometabolites, contribute to carcinogenesis and cancer progression (Yang et al., 2013; Khatami et al., 2019). Among these, 2-hydroxyglutaric acid (2-HG) is the most well-known oncometabolite and 2-HG function has been linked to cancer (Xu et al., 2011). Isocitrate dehydrogenase (IDH) one and two are enzymes that mediate the synthesis of α-ketoglutaric acid (α-KG) from isocitrate, whereas a specific mutation in IDH1/2 results in the production of large amounts of 2-HG from α-KG (Dang et al., 2009). IDH1/2 mutations have been found in glioma, acute myelogenous leukemia (AML), chondrosarcoma, cholangiocarcinoma, and T-cell angioimmunoblastic lymphoma, as well as in colorectal and prostate cancers, with IDH1 R132H and IDH2 R140Q the frequently observed mutations (Ward et al., 2013). High concentrations of 2-HG inhibit α-KG-dependent dioxygenases, including prolyl hydroxylases (PHDs), DNA demethylases (TETs), and histone lysine demethylases (KDMs), which may be involved in cancer progression by causing metabolic reprogramming *via* hypoxia inducible factor (HIF) stabilization and the inactivation of tumor suppressor genes *via* DNA and histone methylation (Yang et al., 2013). However, it is unclear whether 2-HG is present in sEVs released from cells.

In this study, we established a semi-automated method to selectively collect sEVs derived from cultured cells using SEC while monitoring the amount collected. Using the developed SEC method, we recovered sEVs released by IDH1 mutant colon cancer cells and performed metabolomic analysis, which revealed a characteristic metabolomic profile, including high concentrations of 2-HG.

## 2 Materials and methods

### 2.1 Cell culture

The human colon cancer cell line HT29 ectopically expressing CD63-Nanoluc (HT29-CD63-Nluc) was generated at the Aichi Cancer Center Research Institute. This cell line was labeled with the exosome marker CD63 with high-intensity luciferase Nanoluc (Hikita et al., 2018). The human colon cancer cell line HCT116 was obtained from the American Type Culture Collection (ATCC, Manassas, VA, USA), and HCT116 IDH1 (R132H/+) cells were obtained from Horizon Discovery Ltd. (Cambridge, United Kingdom).

HT29-CD63-Nluc cells were grown in RPMI1640 medium (FUJIFILM Wako Pure Chemical Corporation, Osaka, Japan) containing 10% (v/v) Fetal bovine serum (FBS, BioWest, Nuaillé, France) and antibiotics (250 μg/mL amphotericin B, 100 U/mL penicillin, and 100 mg/mL streptomycin, Nacalai Tesque, Kyoto, Japan). HCT116 cell and HCT116 IDH1 (R132H/+) cells were maintained in DMEM high glucose medium (FUJIFILM Wako Pure Chemical Corporation) with 10% (v/v) FBS, antibiotics, and 110 mg/mL sodium pyruvate. All the cells were cultured at 37°C in a humidified atmosphere containing 5% CO_2_. All cells were confirmed to be mycoplasma-free using the Mycoalert detection kit (Lonza, Basel, Switzerland) whenever sEVs were collected.

### 2.2 Collection of cell-cultured medium

HT29-CD63-Nluc cells (3.0 ×10^6^) were seeded into 150-mm dishes and pre-cultured with RPMI1640 containing 10% FBS and antibiotics for 24 h. The cells were washed twice with Dulbecco’s phosphate-buffered saline (D-PBS, Nacalai Tesque). Next, the culture medium was exchanged for advanced RPMI1640 medium (Thermo Fisher Scientific, Waltham, MA, USA) containing 1 μmol/L glutamine (Thermo Fisher Scientific) and antibiotics and cultured for 2 days.

Equal numbers of cells (4.0 ×10^6^) of HCT116 wild-type (WT) cell and HCT116 IDH1 (R132H/+) cell were seeded into 150-mm dishes and pre-cultured with DMEM high glucose medium containing 10% FBS, antibiotics, and sodium pyruvate for 24 h. The cells were then washed twice with D-PBS. Next, the culture medium was exchanged for DMEM high glucose medium with 2% (v/v) exosome depleted FBS (Thermo Fisher Scientific) and antibiotics and cultured for 2 days before the cell-conditioned medium was collected. The cell-culture medium was centrifuged at 2,000 × *g* for 25 min and 15,000 *× g* for 50 min at 4°C to pellet and remove cells, debris, and apoptotic bodies. The supernatants were filtered using a 220-nm polyethersulfone filter (Merck Millipore Ltd., Burlington, MA, USA) to remove the large EVs. The filtrates were concentrated using a 100 kDa cut-off filter (Merck Millipore Ltd.). This suspension was used to prepare the samples.

### 2.3 sEVs collection using size exclusion chromatography

High-performance liquid chromatography (HPLC) was performed using an Agilent infinity 1,200 series (Agilent Technologies, Santa Clara, CA, USA). The samples were injected with 50- or 100-fold enrichment medium. For the separation of sEVs, size-controlled hydrophilic porous silica gel was packed in a 4.6 × 250 mm column (the average particle semidiameter (d50): 7.25 μm, the average pore diameter: 71.8 nm, the pore volume: 1.85 mL/g, and the surface area: 98 m^2^/g) (Yoshitake et al., 2022). The column temperature was maintained at 20°C. The diode array detector (DAD) was monitored at 190–600 nm and fractionated using a wavelength of 204 nm. The mobile phase was composed of D-PBS (A) and MeOH (B; LC-MS grade, FUJIFILM Wako Pure Chemical Corporation). The flow rate of the mobile phase was 0–15.01 at 250 μL/min and 15.01–25.0 at 500 μL/min. The gradient used was .00–10.00 min 0% B; 10.00–10.10 min 0 to 70% B; 10.10–15.00 min 70% B; and 15.00–15.01 min 70 to 0% B, which was maintained until 25.00 min. The collected fractions were adjusted to 50 mg (≈50 μL) using a 100 kDa cut-off filter.

### 2.4 sEVs collection using ultracentrifugation

The concentrate of the cell culture medium was ultracentrifuged at 37,000 rpm (average RCF of 234,700 × *g*) for 70 min at 4°C (SW41Ti rotor and Optima XE-90 Ultracentrifuge, Beckman Coulter, Brea, CA, USA). The pellet was washed with D-PBS and collected *via* ultracentrifugation. This washing procedure was repeated twice. The weight of the pellet was adjusted to 50 mg (≈50 μL) by adding D-PBS.

### 2.5 Extraction of hydrophilic metabolites and lipids from cells

For the measurement of hydrophilic metabolites and lipids, we prepared the extraction solvent, supplemented in 10 μL internal standards mix for lipids [ceramide (Cer) d18:1–17:0, and hexosylceramides (HexCer) d18:1 (d5)-18:1 10 μmol/L; cholesterol (d7), 300 μmol/L; free fatty acid (FFA) 17:0, 500 μmol/L], 10 μL mouse SPLASH^®^ LIPIDOMIX^®^ Mass Spec Standard (Avanti Polar Lipids, Alabaster, AL, USA) containing [lysophosphatidylethanolamine (LPE) 18:1 (d7), 2 μmol/L; phosphatidylglycerol (PG) 15:0–18:1 (d7) and alkenyl-acyl phosphatidylethanolamine (pPE) C18(Plasm)-18:1 (d9), 5 μmol/L; phosphatidylethanolamine (PE) 15:0–18:1 (d7), 7 μmol/L; phosphatidic acid (PA) 15:0–18:1 (d7), 10 μmol/L; diacylglycerol (DAG) 15:0–18:1 (d7), 15 μmol/L; phosphatidylserine (PS) 15:0–18:1 (d7), phosphatidylinositol (PI) 15:0–18:1 (d7), alkenyl-acyl phosphatidylcholine (pPC) C18:0–18:1 (d9), and sphingomyelin (SM) d18:1–18:1 (d9), 20 μmol/L; triacylglycerol (TAG) 15:0–18:1 (d7)-15:0, 35 μmol/L; lysophosphatidylcholine (LPC) 18:1 (d7), 45 μmol/L; phosphatidylcholine (PC) 15:0–18:1 (d7), 100 μmol/L], and 10 μL internal standards mix for hydrophilic metabolites (10-camphorsulfonic acid, L-tryptophan-^13^C_11_-^15^N_2_, and L-methionine sulfone 100 μmol/L) per 1 mL methanol. The cells were washed twice with pre-warmed (37°C) D-PBS and dissolved in 700 μL extraction solvent. The homogenate was then sonicated for 5 min. Then, 400 μL supernatant was mixed with 160 μL methanol, 500 μL chloroform, and 200 μL Milli-Q water (Merck Millipore Ltd.) using a vortex mixer for 1 min, followed by 5 min of sonication. After centrifugation at 16,000 × *g*, 4°C for 5 min, 800 μL supernatant was transferred to a clean tube. Finally, 220 μL chloroform and 220 μL Milli-Q water was added to the supernatant before it was vortexed and centrifuged at 16,000× *g*, 4°C for 5 min.

For hydrophilic analysis, 400 μL supernatant was transferred to clean tubes and lyophilized. Samples were dissolved in 100 μL 50% (v/v) aqueous acetonitrile and immediately used for hydrophilic metabolome analysis.

For lipidomic analysis, 400 μL of the bottom layer was concentrated to dryness under a nitrogen stream and dissolved in 100 μL 50% (v/v) chloroform/methanol.

### 2.6 Extraction of hydrophilic metabolites and lipids from sEVs

Hydrophilic metabolites and lipids were extracted from 45 μL sEVs samples. The extraction solvent (560 μL) was then added to the sEVs. Chloroform (280 μL) and Milli-Q water (160 μL) were added to the samples and mixed vigorously by vortexing, followed by sonication. The subsequent steps were the same as those described for cell preparations. Samples were centrifugated at 16,000 × *g*, 4°C, 5 min and 800 μL supernatant was transferred to a clean tube. This supernatant sample was vortexed and centrifuged at 16,000 × *g*, 4°C for 5 min after adding 220 μL chloroform and 220 μL Milli-Q water.

For hydrophilic analysis, 600 μL supernatant was transferred to clean tubes and lyophilized. Samples were dissolved in 50 μL 50% (v/v) aqueous acetonitrile and immediately used for hydrophilic metabolome analysis.

For lipidomic analysis, 400 μL of the bottom layer was concentrated to dryness under a nitrogen stream and dissolved in 100 μL of 50% (v/v) chloroform/methanol.

### 2.7 Analysis of hydrophilic metabolites and lipids

Anionic metabolites were measured using capillary ion chromatography-mass spectrometry (capillary IC-MS) as previously described (Hirayama et al., 2020). Cationic metabolites were analyzed using liquid chromatography-mass spectrometry (LC-MS) as previously described (Suzuki et al., 2022). Lipids were measured using superfluid liquid chromatography-triple quadrupole mass spectrometry (SFC-QqQMS) as previously described, but with major modifications (Takeda et al., 2018; Hayasaka et al., 2021). The details of the lipid measurement methods are provided in the Supplementary Material.

### 2.8 Data analysis

The data acquired with preparative HPLC were analyzed using ChemStation (version B.04.02, Agilent Technologies). The raw data obtained by capillary IC-MS and LC-MS were analyzed using the TraceFinder software (version 5.0, Thermo Fisher Scientific). The multiple reaction monitoring data acquired using SFC-QqQMS were analyzed using MassHunter software (version 10.0, Agilent Technologies). The metabolite data for the cells and sEVs were subtracted from the value of each blank sample in which no cells were cultured. If the value was negative, it was set to 0. Statistical significance was determined using the Student’s t-test. The Student’s t-test and the coefficient of determination were analyzed using GraphPad Prism (version 8.4.3). Principal component analysis (PCA) and heatmaps were analyzed using JMP (version 16.2.0) and MeV (version 10.2), respectively.

## 3 Results

### 3.1 Development of the sEVs recovery method using semi-automated SEC

**FIGURE 1:**
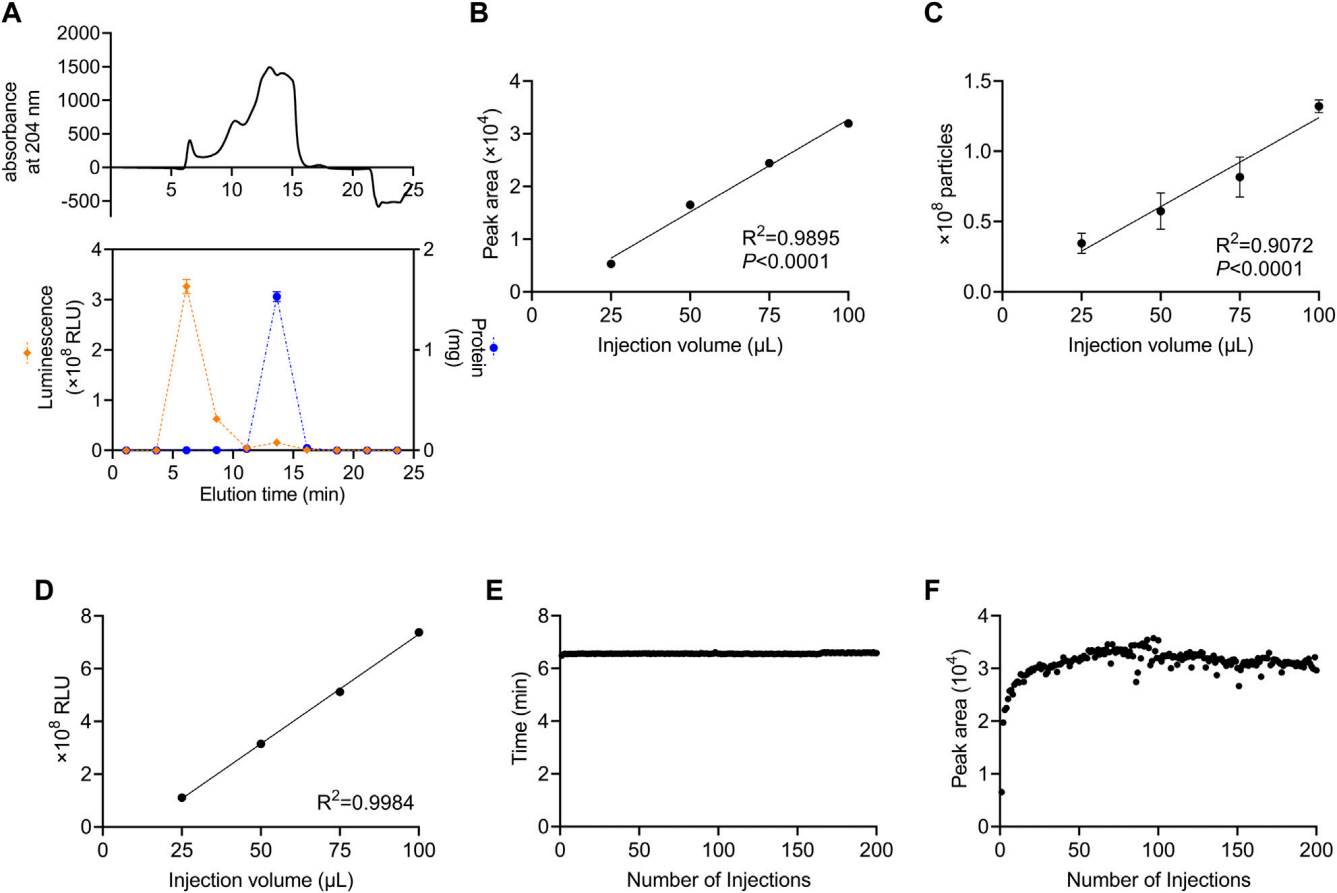
Stable recovery of sEVs derived from cultured cells using SEC **(A)** Isolation of sEVs from cell-cultured medium using the SEC system. Upper panel: the absorption of peptides bind at 204 nm. Lower panel: the Nanoluc luciferase intensity and the protein content of each fraction are represented by orange diamonds (left axis) and blue circles (right axis), respectively. **(B)** Correlation between the injection volume and the peak area of HT29-CD63-Nluc. The peak area was calculated from DAD measurements. **(C)** Correlation between the injection volume and the number of particles measured. The number of particles was measured by NanoSight. **(D)** Correlation between the injection volume and Nanoluc luciferase intensity. The luminescence was measured with a luminometer. **(E)** The time of the peaks from 200 injections. **(F)** The peak area for 200 injections. **(A–D)** data are presented as the means ± SD from triplicate samples.

We developed a method in which a column for SEC was attached to an HPLC system and monitored using a DAD to perform automated preparative separation of sEVs. The column consisted of size-controlled hydrophilic porous silica gel packed into columns for HPLC. First, we investigated which fractions contained sEVs after recovery from culture supernatants using SEC. For sEVs recovery experiments, we used the human colon cancer cell line, HT29-CD63-Nluc, which ectopically expresses Nanoluc, a highly sensitive luciferase fused with CD63, as an sEV marker (Hikita et al., 2018). In these cells, the expression of CD63 was detectable as Nanoluc luciferase intensity. The 100 µL sample of HT29-CD63-Nluc cell culture supernatant as a 50-fold concentration with a 100 kDa filter, was injected and allowed to swim for 30 min, and the peptide binding wavelength, 204 nm, was measured continuously with DAD. While protein absorbance is often measured at 280 nm, in this study we used 204 nm, which is the wavelength at which peptides bind and thus, it is more sensitive. The results showed a peak intensity of approximately 500 mAU after 6.5 min and a significant peak of approximately 1,500 mAU from 12.5 to 16.0 min (Figure 1A). To determine which peak corresponded to sEVs, fractions were sampled every 2.5 min such that 10 fractions were collected. Nanoluc luciferase intensity was observed in the third fraction (5.0–7.5 min), and a large amount of protein was observed in the sixth fraction (12.5–15.0 min) (Figure 1A). We considered the 6.5 min peak in the third fraction to be derived from sEVs. Also, we considered the sixth fraction represents the elution of free protein due to change from 100% D-PBS to 70% Methanol. In subsequent analyses, we established a method to recover the peak portion when a peak was observed between 4.0 and 7.4 min.

Next, we examined whether the number of sEVs correlated with the injection volume. The number of sEVs recovered was compared by injecting 25, 50, 75, and 100 μL culture supernatant concentrates of HT29-CD63-Nluc cells. A strong correlation with the injection volume was observed when the peak area obtained by DAD was calculated (Figure 1B). Corresponding to the area, the number of particles calculated by Nano tracking analysis (NTA) and Nanoluc intensity also showed an injection volume-dependent recovery of sEVs (Figures 1C, D).

The durability of the column was examined by injecting 200 samples of culture supernatant. No considerable differences were observed in either the time or peak areas (Figures 1E, F). This confirmed that sEVs could be stably recovered, even after multiple injections.

### 3.2 The characteristics of sEVs collected using different recovery methods

**FIGURE 2:**
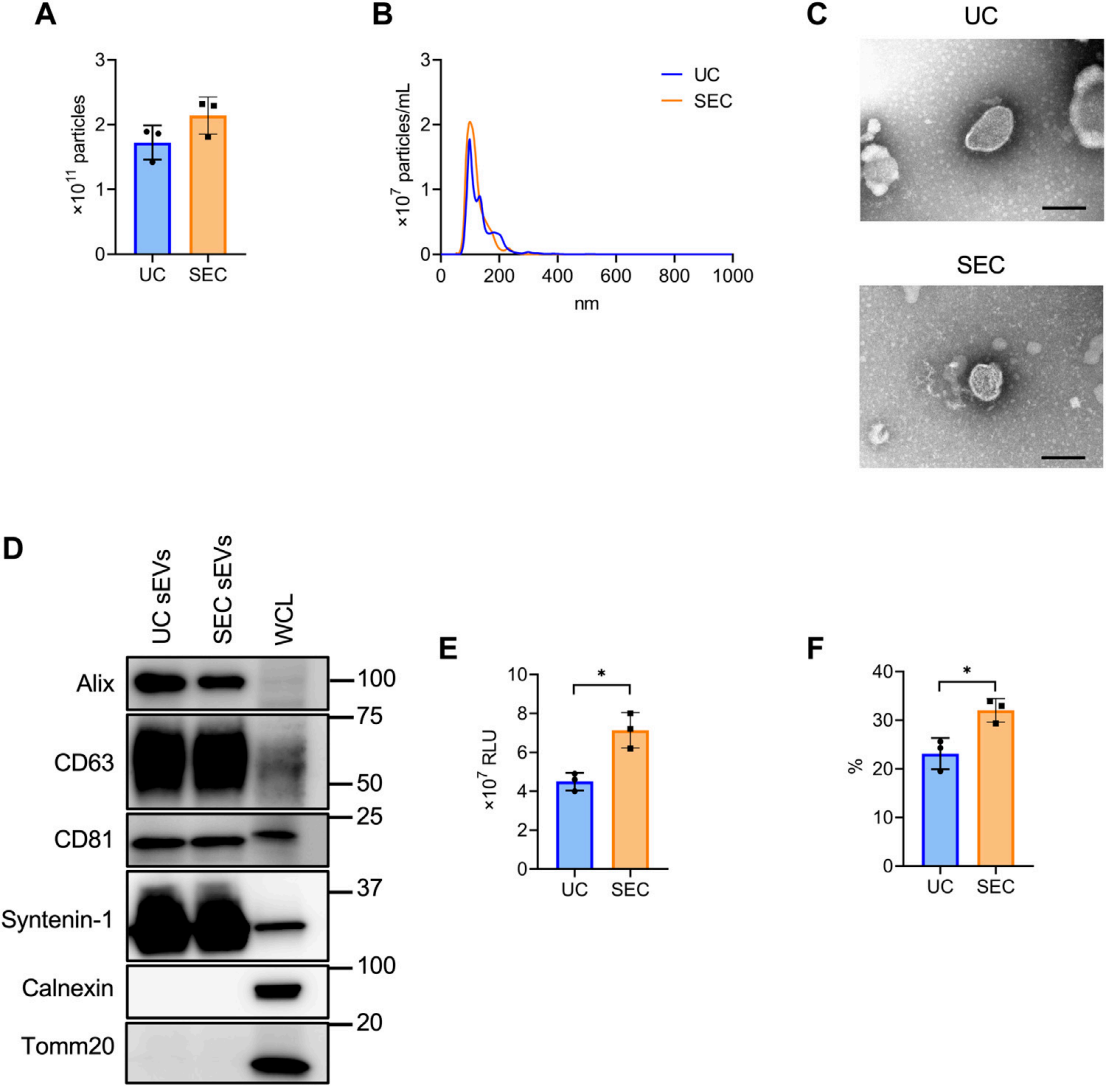
The comparison of sEV collection between UC and SEC. **(A)** The number of particles in UC and SEC samples. **(B)** Size distribution of the particles in UC and SEC samples. **(C)** Transmission electron microscopic images of sEVs using UC and SEC. Scale bar, 100 nm. **(D)** Western blotting analysis of Alix, CD63, CD81, syntenin-1, calnexin, and tomm20 expression in HT29-CD63-Nluc cells and sEVs using UC and SEC recovery systems. The sEV markers, Alix, CD63, CD81, and syntenin-1; sEV negative protein markers, calnexin and tomm20. The protein content was 1.0 μg for the sEV samples and 10 μg for the whole cell lysate (WCL) samples. **(E)** The Nanoluc luciferase intensity of UC and SEC samples. **(F)** The recovery rate of the EVs collected using UC and SEC. The sample after concentration was calculated as 100%. ^*^ *p*-value <.05 by Student’s t-test.

From 200 mL culture supernatant collected from HT29-CD63-Nluc cells, sEVs were isolated by UC and SEC and the quantity and quality were compared. The NTA showed that the average particle size of sEVs recovered by UC and SEC were 135.6 nm and 120.4 nm, respectively, with a smaller particle size recovered by SEC (Figures 2A, B). Transmission electron microscopy (TEM) revealed particles corresponding to sEVs with a lipid bilayer structure (Figure 2C). The sEVs markers Syntenin-1, Alix, CD63, and CD81 were enriched in sEVs, and the endoplasmic reticulum (ER) marker calnexin and mitochondrial marker Tomm20 were absent (Figure 2D). The amount of CD63 present was confirmed by the Nanoluc intensity, which was significantly increased in SEC (Figure 2E). When we calculated the recovery rate with the concentrate as 100%, it was confirmed that significantly more sEVs were recovered with SEC than with UC (Figure 2F).

**FIGURE 3:**
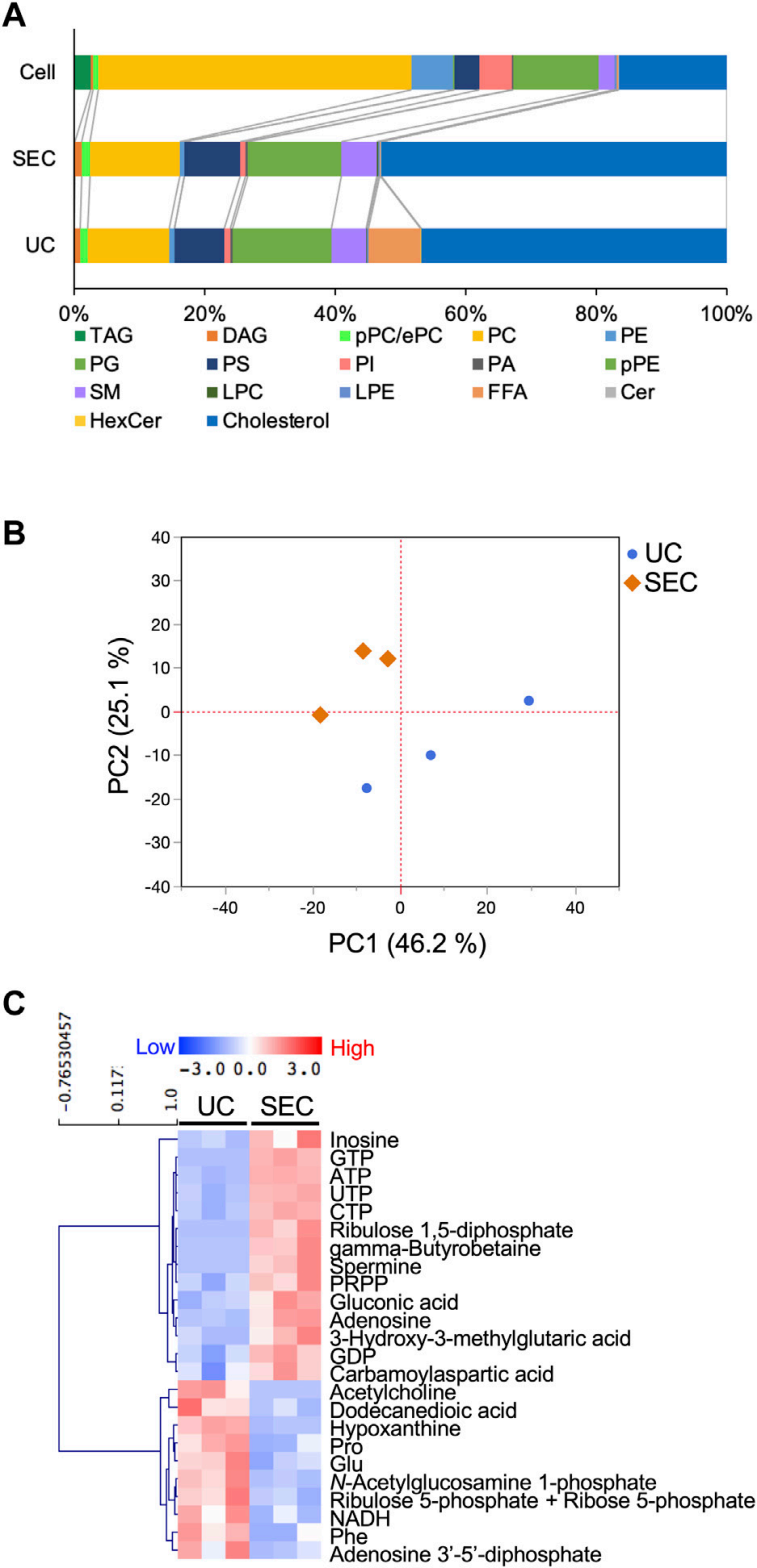
Effect of the collection method on the metabolite profiles in sEVs **(A)** Pie plots showing the mol-based percentile of each lipid class in cells and EVs collected by SEC and UC. **(B)** Principal component analysis (PCA) score plot of hydrophilic metabolites and lipids from UC (blue circles) and SEC (orange diamonds) samples. The contribution ratios were 46.2% and 25.1% for PC1 and PC2, respectively. Metabolites that were detected in over 50% of the samples were included in the analysis. Metabolite data from the non-cultured medium were subtracted and then normalized by particles measured by NanoSight. **(C)** Heatmap showing the metabolite profiles of sEVs that significantly changed metabolites between UC and SEC recovery systems. The *p*-value was calculated using the Student’s t-test, and *p*-value < .05 was considered a significant change. *n* = 3.

We measured hydrophilic metabolites using capillary IC-MS and LC-MS, and lipids using SEC-QqQMS, from both sEVs and cell samples. When analyzing metabolites in sEVs, it is necessary to control the effects of the large amounts of metabolites present in uncultured media. Therefore, we prepared blank sEV samples for this study. Uncultured medium was incubated in a CO_2_ incubator and processed with UC or SEC to prepare sEVs blank samples. A total of 586 metabolites (109 hydrophilic metabolites and 477 lipids) and 595 metabolites (115 hydrophilic metabolites and 480 lipids) were identified in sEVs from culture supernatants derived from HT29-CD63-Nluc cells recovered with UC and SEC, respectively. The common metabolites in the samples retrieved with UC and SEC were 540 (81 hydrophilic metabolites and 459 lipids). A relationship between the lipid profiles of sEVs and cells common to many cells has been reported (Llorente et al., 2013; Nishida-Aoki et al., 2020; Skotland et al., 2020). In the present study, we examined whether a similar relationship between cell and sEV lipid compositions could be measured. Cholesterol, PS, and SM were enriched, whereas PC and PI were not. Compared to cells, cholesterol (UC 2.8 times, SEC 3.2 times), PS (UC 2.0 times, SEC 2.2 times), and SM (UC 2.2 times, SEC 2.2 times) were enriched in sEVs, while PC (UC .3 times, SEC .3 times) and PI (UC .2 times, SEC .2 times) were not. Similar changes were observed, as in a previous study (Llorente et al., 2013; Nishida-Aoki et al., 2020; Skotland et al., 2020), suggesting that both UC and SEC could measure sEV-specific lipids (Figure 3A). When analyzed by lipid class, FFA were detected only in UC; otherwise, there was no difference in the classes detected between the UC and SEC methods (Figure 3A). For metabolites overall, the PCA showed a separation of plots between UC and SEC, suggesting metabolite profiles in sEVs differ depending on the recovery method used (Figure 3B). When we focused on hydrophilic metabolites that varied significantly between UC and SEC, those involved in purine-pyrimidine metabolism, such as GTP, ATP, UTP, CTP, inosine, adenosine, and GDP, were abundant in SEC; while in contrast, amino acids such as Pro, Glu, and Phe were abundant in UC (Figure 3C). We found that 20 lipids were significantly increased in UC when compared to SEC, including three types of TAG, three types of PE, three types of LPE, and four types of HexCer. In comparison, a total of 42 lipids were significantly increased in SEC, including 11 TAG, 12 PC, 12 LPC, and six SM (Table 1).

**TABLE 1:** Number of lipids that significantly varied between UC and SEC recovery methods.

Lipid	UC	SEC
Triacylglycerol (TAG)	3	11
Diacylglycerol (DAG)	0	1
Phosphatidylcholine (PC)	1	12
Phosphatidylethanolamine (PE)	3	0
Phosphatidylglycerol (PG)	2	0
Alkenyl-Acyl Phosphatidylethanolamine (pPE)	1	0
Lysophosphatidylcholine (LPC)	2	12
Lysophosphatidylethanolamine (LPE)	3	0
Sphingomyelin (SM)	0	6
Ceramide (Cer)	1	0
Hexosylceramides (HexCer)	4	0
Free Fatty Acid (FFA)	0	0
Total	20	42

*p-value <.05 by Student’s t-test.

### 3.3 Metabolome analysis of sEVs isolated from IDH1-mutated HCT116 cells using the SEC method

**FIGURE 4:**
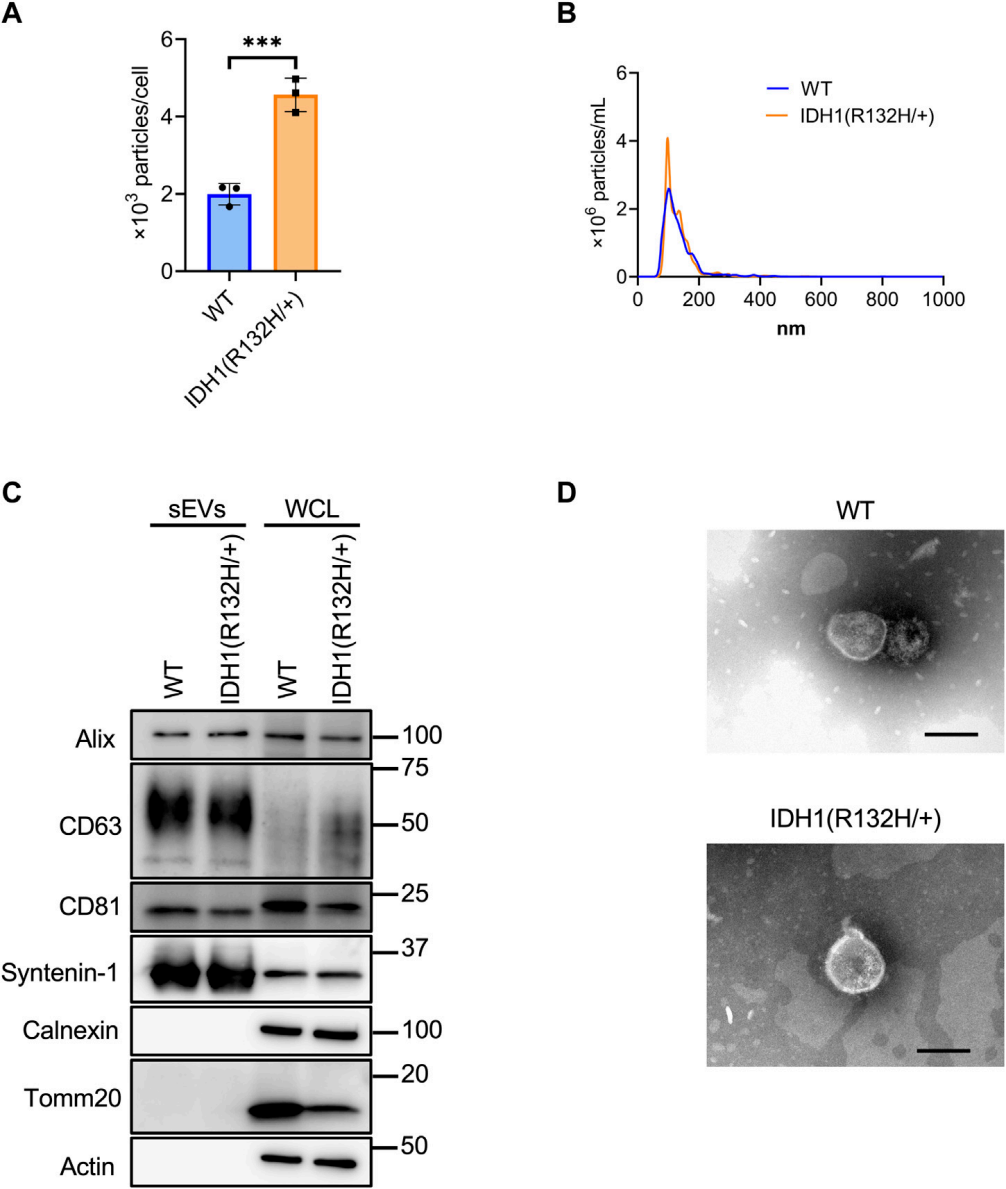
Characterization of sEVs derived from HCT116 wild-type and HCT116 IDH1(R132H/+) cell lines. **(A)** The number of particles from sEVs derived from HCT116 wild-type (WT) and IDH1 (R132H/+) cell lines. ***p-value <.001 by Student’s t-test. **(B)** Particles distribution of sEVs from HCT116 WT and IDH1(R132H/+) cells. **(C)** Western blotting analysis of Alix, CD63, CD81, syntenin-1, calnexin, tomm20, and actin, which was used as a loading control. The injection amount was 1.0 μg for the sEV sample and 10 μg for the WCL sample. **(D)** Transmission electron microscopic images of sEVs derived from HCT116 WT and IDH1(R132H/+) cells. Scale bar, 100 nm.

Using the developed SEC method, sEVs were collected from 200 mL culture supernatants from the wild-type and IDH1 mutant (R132H/+) strains of the colon cancer cell line HCT116. Significantly more sEVs were recovered from the IDH1 mutant cells than WT cells (Figure 4A). The mean particle size was the same for the IDH1 mutant cells and WT cells at about 135 nm. (Figure 4B). Western blotting analysis detected the sEVs marker proteins Syntenin-1, Alix, CD63, and CD81, respectively, and the non-marker proteins calnexin and Tomm20, respectively, in the collected particle samples (Figure 4C). TEM analysis revealed particles with a diameter of approximately 100 nm in vesicular structures in both wild-type and mutant cells (Figure 4D).

**FIGURE 5:**
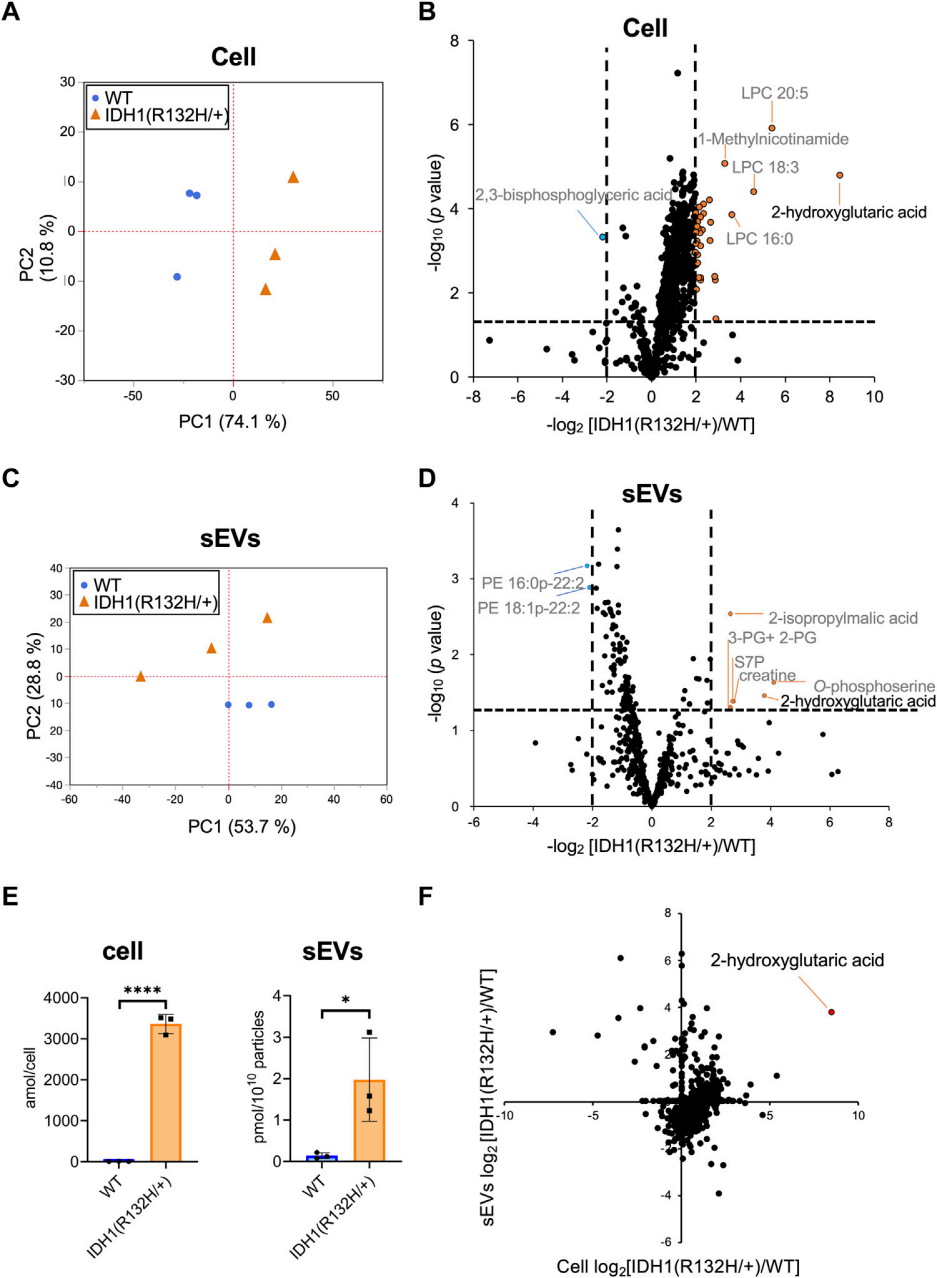
Effects of IDH1(R132H/+) mutations on metabolites in cells and extracellular vesicles **(A)** Principal component analysis (PCA) score plots between wild-type (WT, blue circles) and IDH1(R132H/+) (orange triangles) cells. **(B)** The result of a Student’s t-test for WT EVs vs IDH1(R132H/+) is displayed as a volcano plot. The orange and blue plots are for the metabolites that increased and decreased between WT and IDH1(R132H/+), respectively. *p*-value <.05, fold-change >4. **(C)** PCA score plots between WT (blue circles) and IDH1(R132H/+) (orange triangles) in sEVs. **(D)** The volcano plots illustrate the quantitative differences in metabolites in the sEV fraction. The plots marked orange and blue are same as **(B)**. **(E)** Levels of 2-hydroxyglutarate in cells and EVs. Data are represented as mean ± SD. *p-value < .05, **p-value < .001. **(F)** The relationship between cellular and sEVs variability. The x- and *y*-axes indicate the variation in cell and sEVs, respectively. Variation was calculated as the ratio of IDH1 (R132H/+) to WT.

Hydrophilic metabolomic analysis using capillary IC-MS, LC-MS, and lipid analysis using SFC-QqQMS were performed on sEVs and cell samples. When metabolites were measured in IDH1-mutated HCT116 cells, 900 were identified in more than 50% of the samples, including 185 hydrophilic metabolites (102 by capillary IC-MS and 83 by LC-MS) and 715 lipids. PCA revealed segregation in principal component 1, indicating that IDH1 mutations altered the cellular metabolite profile from that of the wild-type (Figure 5A). IDH1 mutations in cancer cells result in the production of large amounts of 2-HG (Xu et al., 2011), and the volcano plot shows that 31 metabolites, including 2-HG, were increased (*p*-value <.05, fold-change >2), and a significant decrease was observed only for 2,3-bisphosphoglyceric acid when compared with that in the WT cells (Figure 5B). As in the previous study (Dang et al., 2009), the IDH1 mutation cell line resulted in a high production of 2-HG in this experiment.

Metabolite profiles in sEVs with and without mutations in the IDH1 mutation were measured. The metabolite values obtained from the non-cultured medium were subtracted from those of the sEVs samples and showed a total of 660 metabolites of which 85 were hydrophilic (64 by capillary IC-MS and 21 by LC-MS) and 575 were lipids in sEVs in more than 50% of samples. PCA was performed on metabolites that comprised more than 50% of the total metabolites, which showed a separation of the second principal component and different metabolite profiles (Figure 5C). As for metabolites in sEVs, in addition to 2-HG, 2-isopropylmalic acid, 3-phosphoglyceric acid (3-PG) +2-phosphoglyceric acid (2-PG), sedoheptulose 7-phosphate (S7P), creatine, and *O*-phosphoserine were increased in sEVs derived from the IDH1 mutation cell line, and PE 16:0p-22:2 and PE 18:1p-22:2 decreased (*p*-value < .05, and less than one-fourth, Figure 5D). Therefore, we analyzed whether the cellular 2-HG content affected the sEV 2-HG content. The results showed that 2-HG levels were significantly increased in the mutant cells and in the sEVs isolated from the IDH1 mutation cell line when compared with those of WT cell line (Figure 5E). Finally, we examined the extent to which the variation between WT and IDH1 mutant cell lines observed in cells was reflected in sEVs (Figure 5F). In sEVs, only 274 of the 660 metabolites showed the same variation as that in cells, whereas the remaining 352 metabolites showed different variations. The metabolites commonly increased in cells and sEVs included 2-HG and other metabolites in the TCA cycle, such as citric acid, cis-aconitic acid, isocitric acid, succinic acid, fumaric acid, malic acid; as well as metabolites involved in the glycolytic system, such as G6P and F6P. Together, these results showed that the metabolite profile of the extracellular vesicles do not fully reflect the change in the cells’ metabolomic profile due to IDH1 mutant. Nevertheless, extracellular vesicles derived from IDH1 mutant included 2-HG.

## 4 Discussion

sEVs are involved in intercellular communication and cancer progression, and have attracted much attention (Raposo and Stoorvogel, 2013). One of the bottlenecks in sEVs research is the available collection methods for sEVs. For the further development of sEVs, a method must be efficient in recovering sEVs, suppressing foreign substances outside the sEVs, and cost- and time-efficient (Veerman et al., 2021). In addition, a method must be able to recover extracellular vesicles with minimal damage to the MS equipment for metabolomic and proteomic analyses. To solve these problems, we developed a method to fractionate and automatically collect extracellular vesicles using size-exclusion chromatography and examined their effects on metabolites.

SEC has been used to recover sEVs from urine, blood, and cultured cell supernatants (Sidhom et al., 2020). Most of the SEC protocols are kit-based, such as qEV and EV Second, and manual recovery of sEVs or kit-specific equipment are limited in their versatility (Sun and Meckes, 2018). In this experiment, sEVs derived from the culture supernatant were automatically collected within 30 min per injection using an HPLC column packed with size-controlled hydrophilic porous silica gel. Injection volume-dependent changes in the area, particle count, and CD63-Nanoluc of the concentrated cell culture supernatant were observed, indicating that we successfully achieved injection volume-dependent collection of sEVs. Calculating the area at 204 nm using DAD may be a used as a rough indicator for monitoring the recovery of sEVs. The time deviations were minimal, except for the first injection; however, the sEVs were stably recovered 200 times. Perhaps the solution is to perform a trial run before the first collection of sEVs and mask the adsorption with the column. UC and various kits have been problematic because of the reported effects on sEVs recovery (Brennan et al., 2020). Because the present method can set the sample and collect the sEVs, it may be possible to collect the sample with less user-dependent variation.

A comparison of sEVs recovery by SEC and UC revealed that both methods enriched the sEVs markers Alix, CD63, CD81, and Syntenin-1. Neither the ER marker calnexin nor the mitochondrial marker Tomm20 were detected. Additionally, sEVs with intact lipid bilayer structures were recovered. Compared to the UC method, particles with smaller diameters were recovered to the same extent by SEC, and significantly more particles were recovered that expressed the CD63-Nluc marker for sEVs. In addition, some of the products may have been denatured and not detected because methanol was used for cleaning. This phenomenon has been reported in similar studies using SEC (Takov et al., 2017; Takov et al., 2019; Veerman et al., 2021). In this case, we prioritized purity and narrowed the recovery time. Nevertheless, we may be able to expect further improvement in the recovery rate by delaying the end time of recovery slightly longer. However, in studies using SEC on urine to recover fractions slower than the sEV fractions, we observed different protein profiles as the particles became smaller and slower (Guan et al., 2019). Because they may not be the target sEVs, careful consideration is necessary to determine whether they should be included in the recovery fraction.

Previous studies have used SEC to recover sEVs for metabolomic analyses (Ludwig et al., 2020; Du et al., 2021). Although different methods of sEVs recovery can result in different sEVs protein and RNA profiles, their effect on metabolites is unknown. Therefore, we compared the metabolomic analysis of sEVs recovered using ultracentrifugation as the gold standard to evaluate SEC. First, the non-cultured medium was subjected to the same process as those used for the recovery of sEVs, and metabolomic analysis identified 324 metabolites (100 hydrophilic metabolites and 224 lipids) by ultracentrifugation, and 250 metabolites (107 hydrophilic metabolites and 143 lipids) by SEC, indicating that the ultracentrifugation method is likely to detect more noise. Known contaminants during pretreatment and measurement include plasticizers, solvent impurities, reagents used during sample cleanup, and carryover contamination (Broadhurst et al., 2018). Martínez-Sena’s paper reported that the blank sample detected 2843 UPLC-MS signals (76% of the total) as noise (Martínez-Sena et al., 2019). In this study, these metabolites may have also originated from the culture medium, process of sEVs collection, pretreatment, or measurement. Since these metabolites were derived from sources other than sEVs, the response was to subtract them as blanks from the sample. When performing metabolite analysis of sEVs, it may be necessary to include an appropriate blank sample to determine the extent to which foreign substances affect the analysis.

In a study comparing the effects of different sEV collection methods on RNA in plasma, SEC was optimal because the miRNA-binding protein Argonaute-2 (AGO-2) was the lowest, EV-specific miRNA and lncRNA were observed most frequently, and non-specific miRNA was the lowest (Yang H et al., 2021). In a study comparing the effect of different methods of sEV recovery on protein in urine, SEC reported better recovery of sEVs and purification in terms of protein (Guan et al., 2020). We also detected metabolite contamination of non-EVs using the UC method. Noise was present in the actual sample, as well as in the non-cultured medium, suggesting that the SEC method can recover the sample with a better degree of purification. Therefore, in this study, quantitative values were obtained by subtracting each of the metabolites detected in the non-cultured medium using the corresponding method from the actual samples. However, only advanced RPMI 1640 medium was used as the uncultured medium sample in this study, and a different medium may produce different results.

Lipid analysis of sEVs from cultured cells has shown that PS, cholesterol, and SM are enriched, and PC and PI are lower in sEVs than in cells (Llorente et al., 2013; Nishida-Aoki et al., 2020; Skotland et al., 2020). Similar changes were observed in this study when comparing the sEVs recovered by ultracentrifugation and SEC with the lipid class on the releasing cells. It is believed that the sEVs were successfully recovered at the lipid class level, with FFA the only lipid that showed significant variation between UC and SEC. In the non-cultured medium that was subtracted as a blank, the FFA content was higher in SEC than in UC, suggesting this lipid was not observed in SEC because of the non-cultured medium was subtracted as background in the analysis. The sEVs recovered by SEC and UC had different metabolite profiles. Specifically, the hydrophilic metabolites that varied significantly included those involved in purine-pyrimidine metabolism increased dramatically in SEC. Several metabolites involved in purine-pyrimidine metabolism, including uridine, uracil, and adenosine, have been reported in the breast cancer cell line derived sEVs (Tadokoro et al., 2020). Ludwig et al. identified many metabolites, including purines (Ludwig et al., 2020). Furthermore, in our previous study, the metabolites involved in purine-pyrimidine metabolism were more likely to be found in sEVs (Hayasaka et al., 2021). In many studies, hydrophilic metabolites involved in purine-pyrimidine metabolism have been frequently observed in sEVs (Ludwig et al., 2020; Tadokoro et al., 2020; Hayasaka et al., 2021), and many could be detected when recovered by SEC. In contrast, the UC method detected significantly more amino acids such as Pro, Glu, and Phe than SEC. These amino acids are also present in large amounts in the medium and may have accumulated under the medium influence. Focusing on the lipids that were significantly altered, a significant increase in PC, LPC, and TAG was observed in SEC and SM, suggesting they are enriched in sEVs. PCs and LPCs are known to be limited in sEVs, but many reports indicate that they are present (Skotland et al., 2019; Nishida-Aoki et al., 2020). TAG is a lipid that, along with CE, is abundant in HDL, LDL, and VLDL (Sun et al., 2019). In a study by Nishida et al. using the same medium as in this study, the authors suggested that TAG and CE may be residues from the medium and affect sample processing (Nishida-Aoki et al., 2020), which is a potential interpretation of the results observed in this study. There may be some identically sized contamination, but this is unlikely since the mitochondrial marker Tomm20 and the ER marker calnexin were not observed. No lipid class-specific changes were observed using the UC method.

We used the IDH1 mutation as a way to assess the application of our sEV recovery system. Previous studies have reported that in cells, mutations in IDH1 produce large amounts of 2-HG, which has oncogenic functions (Ward et al., 2013). In this study, sEVs were also recovered within the marker and particle size distribution, and the IDH1 mutation cells released significantly more sEVs than the WT cells. Similar results have been reported in studies of mutant strains using glioma cells (Ludwig et al., 2022). In intrahepatic cholangiocarcinoma, a mutation in IDH1 (R132C) suggested that the downregulation of *P2RX7* was associated with the release of sEVs (Zhang et al., 2019), further supports that mutations in IDH1 promote the release of sEVs. We also found that the IDH1 mutation altered the metabolic profile of sEVs and significantly increased 2-HG levels in sEVs and cells. The IDH1 mutation cell line released a large number of sEVs and although the amount of 2-HG per sEVs was limited, they may carry more 2-HG to recipient cells.

Yang Y et al. (2021) reported that 5-fluorouracil (5-FU)-resistant cell lines secrete sEVs containing high levels of the IDH1 protein, which enhances 5-FU resistance. Indeed, 2-HG is involved in suppressing antitumor T-cell immunity (Bunse et al., 2018) and IDH1 mutation cell line-derived sEVs may also be involved in immunosuppression. It has also been reported that 2-HG is not readily taken up by cells and that 2-HG added to the culture medium is not intracellularly metabolized in colon cancer cell lines (Gelman et al., 2015). Therefore, additional analyses are needed to clarify if 2-HG is present in sEVs and transported to other cells.

Using the pancreatic cancer cell line PANC-1, we previously reported that the metabolite profiles of sEVs were distinct from the cells (Hayasaka et al., 2021). The present study observed similar intracellular metabolic variations in sEVs isolated from IDH1 mutation cells for only 41.5% of the metabolites identified, indicating that the intracellular metabolite fluctuations were not directly reflected in the metabolite profiles of sEVs. Among the metabolites with the same variation in cells and sEVs was 2-HG, which may be encapsulated in sEVs.

This study had several limitations. Although SEC was used to recover sEVs in this study, only the sEVs derived from cultured cells were used and results may be different from sEVs recovered from blood or urine samples. Because of the limitations of the HPLC system, only a total volume of 100 µL could be injected; therefore, multiple injections had to be performed. We believe that the SEC collection system can further shorten the sEVs collection time by improving the injection syringe and adjusting the column thickness and length to accommodate larger sample volumes. Owing to limitations of the experimental facilities, each of the experiments including in this study involved one cancer cell line, and the results may differ for other cell lines.

## 5 Conclusion

We constructed a semi-automated system to collect sEVs by SEC. The recovery of sEVs by SEC was more efficient than that by UC, and metabolomic analysis using capillary IC-MS, LC-MS, and SFC-QqQMS revealed significantly higher concentrations of purine pyrimidine metabolic intermediates. Furthermore, recovery using the constructed recovery system revealed variable metabolite profiles in sEVs depending on the presence or absence of IDH1 mutations, and loading of 2-HG in the oncometabolite was also observed. Thus, sEVs may carry metabolites involved in cancer progression.

## Data Availability

The original contributions presented in the study are included in the article/Supplementary Material, further inquiries can be directed to the corresponding author.
