# Supplementary material for: Metabolomics of small extracellular vesicles derived from isocitrate dehydrogenase 1-mutant HCT116 cells collected by semi-automated size exclusion chromatography
Source: Front Mol Biosci. 2023 Jan 11;9:1049402. doi: 10.3389/fmolb.2022.1049402 (PMC9873957; doi:10.3389/fmolb.2022.1049402)
Supplement: Supplementary file 1 [file Table1.pdf]

# Supplementary Material

## 1 Materials and Methods

### 1.1 Nanoluc assay

The collection sample were diluted to 50 µL using Dulbecco's phosphate-buffered saline (D-PBS, Nacalai Tesque, Kyoto, Japan) and transferred to the white-walled 96 well plates. 50 µL of Nano-Glo substrate diluted 1:50 provided buffer (Nano-Glo Luciferase Assay System, Promega, Madison, WI, USA) were added. Nanoluc luciferase intensity were measured by infinite M200 ABS-FL (Tecan, Männedorf, Switzerland).

### 1.2 Protein quantification of sEVs

The collection sample were diluted using D-PBS, 10 µL of m-PER buffer added. The sample were reacted with microBCA Protein Assay Kit (Thermo Fisher Scientific, Waltham, MA, USA) for 2 h at 37°C. The wavelengths of 562 nm was measured using infinite M200 ABS-FL. The protein content was measured by constructing a calibration curve with 8 points ranging from 0 to 40 µg/mL.

### 1.3 Nanoparticle Tracking Analysis (NTA)

We determined about the number of particle and size distribution of particles by using Nanoparticle Tracking Analysis (NanoSight LM10, NanoSight Ltd., Salisbury, England). Three 60 s measurements were recorded for each sample. The camera level was set at 14 and the detection threshold at 7.

### 1.4 Western blotting

Western blotting analysis was conducted as previously described (Hayasaka et al., 2021). The amount of protein injected is 1.0 µg for sEVs and 10 µg for cells. The following antibodies were used: anti-Alix (#2171, Cell signaling technology, Danvers, MA, USA), anti-CD63 (ab8219, Abcam, Cambridge, UK), anti-CD81 (ANC-302-020, Ancell corporation, Stillwater, MN, USA), anti-Syntenin-1 (ab133267, Abcam), anti-Calnexin (#2679, Cell signaling Technology), and anti-Tomm20 (WH0009804M1-100UG, Merck KGaA, Darmstadt, Bundesrepublik Deutschland). HRP-labeled anti-mouse IgG antibody (NA931-100UL, Cytiva, Marlborough, MA, USA) and anti-rabbit IgG antibodies (NA934-100UL, Cytiva) were used as the secondary antibodies. Results were generated using Clarity Western ECL Substrate (Bio-Rad Laboratories, Hercules, CA, USA) or Carity Max Western ECL Substrate (Bio-Rad Laboratories).

### 1.5 Transmission Electron Microscope (TEM)

Transmission electron microscopy (TEM) analysis was conducted by Tokai Electron Microscopy, Inc (Aichi, Japan) as described previously (Hayasaka et al., 2021).

### 1.6 Lipidomic analysis

Lipids were measured by Supercritical Fluid Chromatograph - Triple Quadrupole Mass Spectrometer (SFC-QqQMS), as previously described but with major modifications (Hayasaka et al., 2021, Takeda et al., 2018). SFC-QqQMS analysis was performed using an Agilent 1260 InfinityII SFC system equipped with an Agilent 6470A triple quadrupole LC/MS system with

Agilent jet stream (AJS) Electrospray ionization (ESI) interface (Agilent Technologies, Santa Clara, CA, USA). The injection volume was 2.0 µL, and the column temperature at 60 ◦C. The mobile phase consisted of supercritical carbon dioxide (A) and methanol/water (95/5, v/v) with 0.1% (w/v) ammonium acetate (B). Analysis of free fatty acid (FFA), diacylglycerol (DAG), triacylglycerol (TAG), ceramide (Cer), hexosylceramides (HexCer), phosphatidylethanolamine (PE), and alkenyl-acyl phosphatidylethanolamine (pPE) were separated on a Viridis HSS C18 SB column (3.0 × 100 mm, 1.8 µm; Waters, Milford, MA, USA). The flow rate of the mobile phase was 1.0 mL/min, and the gradient of solution B was as follows: 1% at 0 min, 50% at 25.0 min, 50% from 25.0 to 28.0 min, and 0% at 28.1 min; this was maintained until 30 min. Analysis of cholesterol, phosphatidylcholine (PC), alkenyl-acyl phosphatidylcholine (pPC)/ alkyl-acyl phosphatidylcholine (ePC), sphingomyelin (SM), lysophosphatidylcholine (LPC), lysophosphatidylethanolamine (LPE), phosphatidylglycerol (PG), phosphatidylinositol (PI), phosphatidylserine (PS), and phosphatidic acid (PA) were separated on a Torus diethanolamine (DEA) column (3.0 × 100 mm, 1.7 µm; Waters). The flow rate of the mobile phase was 1.0 mL/min, and the following linear gradient was used: 0–1.0 min, 1% B; 1.0–24.0 min, 1 to 75% B; 24.0–26.0 min, 75% B; 26.0–26.4 min, 70 to 1% B; this was maintained until 30 min.

AJS-ESI-MS/MS analysis was performed in a positive and negative ion mode, and the conditions were as follows: dry gas temperature = 300 ◦C, dry gas flow rate = 10 L/min, nebulizer pressure = 30 psi, sheath gas temperature = 350 ◦C, sheath gas flow rate = 12 L/min, capillary voltage = 3.0 kV, nozzle voltage = 0 V, fragmentor voltage = 380 V. Data were acquired using dynamic multiple reaction monitoring (MRM) mode, and the MRM transition setting was based on the in-house lipid MRM library described previously (Takeda et al., 2018).

## References

Hayasaka, R., Tabata, S., Hasebe, M., Ikeda, S., Ohnuma, S., Mori, M., et al. (2021). Metabolomic analysis of small extracellular vesicles derived from pancreatic cancer cells cultured under normoxia and hypoxia. *Metabolites* 11, 215. doi:10.3390/metabo11040215

Takeda H., Izumi Y., Takahashi M. Paxton, T. Tamura, S. Koike, T. et al. (2018). Widely targeted quantitative lipidomics method by supercritical fluid chromatography triple quadrupole mass spectrometry. *J. Lipid Res.* 59, 1283–1293. doi:10.1194/jlr.D083014
